# Imaging Correlates between Headache and Breast Cancer: An [^18^F]FDG PET Study

**DOI:** 10.3390/cancers15164147

**Published:** 2023-08-17

**Authors:** Lidija Antunovic, Alessia Artesani, Alessandro Viganò, Arturo Chiti, Armando Santoro, Martina Sollini, Silvia D. Morbelli, Rita De Sanctis

**Affiliations:** 1 IRCCS Humanitas Research Hospital, Via Manzoni 56, 20089 Rozzano, Italy; antunovic.lidija@hsr.it (L.A.); alessia.artesani@hunimed.eu (A.A.); chiti.arturo@hsr.it (A.C.); armando.santoro@cancercenter.humanitas.it (A.S.); rita.de_sanctis@hunimed.eu (R.D.S.); 2 Department of Biomedical Sciences, Humanitas University, Via Rita Levi Montalcini 4, 20072 Pieve Emanuele, Italy; 3 IRCCS Fondazione Don Carlo Gnocchi, 20148 Milan, Italy; avigano@dongnocchi.it; 4 Nuclear Medicine Unit, IRCCS Ospedale Policlinico San Martino, 16132 Genoa, Italy; silviadaniela.morbelli@hsanmartino.it; 5 Department of Health Sciences, University of Genoa, 16132 Genoa, Italy

**Keywords:** primary headache, [^18^F]FDG PET/CT, brain metabolism, neoadjuvant chemotherapy, breast cancer, treatment response, hormonal status

## Abstract

**Simple Summary:**

[^18^F]Fluorodeoxyglucose ([^18^F]FDG) positron emission tomography (PET) provides information about metabolic patterns of different diseases and conditions. This study aimed to prospectively evaluate patients with breast cancer in order to describe specific brain metabolic patterns related to the presence or absence of primary forms of headache, namely tension-type headache (TTH) and migraine (MiG). Moreover, we explored the association between primary headache forms and BC response to neoadjuvant chemotherapy (NAC). We observed a high rate of headache in the 46 BC analyzed patients. TTH patients exhibited areas of hypometabolism in specific brain regions before NAC. Moreover, our results suggest an association between primary headache, especially MiG, and treatment response to NAC. Collectively, our results support the hypothesis of a complex and dynamic interplay among BC, headache, and hormonal status.

**Abstract:**

This study aimed to examine brain metabolic patterns on [^18^F]Fluorodeoxyglucose ([^18^F]FDG) positron emission tomography (PET) in breast cancer (BC), comparing patients with tension-type headache (TTH), migraine (MiG), and those without headache. Further association with BC response to neoadjuvant chemotherapy (NAC) was explored. In this prospective study, BC patients eligible for NAC performed total-body [^18^F]FDG PET/CT with a dedicated brain scan. A voxel-wise analysis (two-sample *t*-test) and a multiple regression model were used to compare brain metabolic patterns among TTH, MiG, and no-headache patients and to correlate them with clinical covariates. A single-subject analysis compared each patient’s brain uptake before and after NAC with a healthy control group. Primary headache was diagnosed in 39/46 of BC patients (39% TTH and 46% MiG). TTH patients exhibited hypometabolism in specific brain regions before NAC. TTH patients with a pathological complete response (pCR) to NAC showed hypermetabolic brain regions in the anterior medial frontal cortex. The correlation between tumor uptake and brain metabolism varied before and after NAC, suggesting an inverse relationship. Additionally, the single-subject analysis revealed that hypometabolic brain regions were not present after NAC. Primary headache, especially MiG, was associated with a better response to NAC. These findings suggest complex interactions between BC, headache, and hormonal status, warranting further investigation in larger prospective cohorts.

## 1. Introduction

Headache is one of the most common nervous system disorders. People of any age, race, and geographic provenience could suffer from headache attacks. More frequent headache forms affect about 35% of the world’s adult population [1].

The third edition of the International Classification of Headache Disorders (ICHD-3) categorizes headaches into primary and secondary forms [2]. Primary headaches include tension-type headache, migraine, trigeminal autonomic cephalalgias, and other minor primary headache disorders. The most common primary form is the tension-type headache (TTH) characterized by pressing or tightening, non-pulsating, mild or moderate pain localized bilaterally, which is not worsened with routine physical activity—neither is it accompanied by nausea or vomiting. In contrast, a migraine (MiG) is defined as pulsating, moderate or severe, unilateral pain that often worsens or hinders routine physical activity; the majority of patients experience accompanying symptoms such as nausea and/or vomiting, photophobia, or phonophobia during migraine episodes. Secondary headache forms arise as a result of an underlying condition that triggers pain in the cranial region [2].

Although headache imaging is mainly performed to exclude secondary forms [3], numerous studies have explored the pathological pathways and morphological changes associated with headache, mainly by means of magnetic resonance (MRI) [4,5,6,7,8,9]. Limited evidence of functional brain changes has been reported using [^18^F]Fluorodeoxyglucose ([^18^F]FDG) positron emission tomography (PET) [3,4,10,11,12,13]. Previous studies have identified regions of relative hypometabolism in MiG patients, including the bilateral insula and cingulate cortex, left premotor and prefrontal cortex, and left visual and left primary somatosensory cortex [10]. Hypometabolism in the frontal and temporal cortex compared to controls has also been reported in patients with chronic MiG [12]. Furthermore, different hypometabolic patterns have been observed in patients with episodic versus chronic MiG [13]. Despite these initial findings, the role of [^18^F]FDG PET in the clinical work-up of MiG patients remains undefined, and its pathophysiological significance requires further clarification.

The frequency of headache episodes in females has been linked to the fluctuation of estrogen levels throughout the reproductive cycle [14]. Higher lifetime exposure to estrogen is associated with an increased risk of breast cancer (BC) development, particularly in individuals with an earlier age of menarche [15] or a late cessation of ovarian function [16]. Certain drugs, including some antineoplastic agents, may influence headache onset [17]. MiG patients undergoing cancer treatment have reported an increased frequency of occasional episodes [18]. Although both headache and BC are tightly related to female hormonal levels, the interaction between these two conditions has not been fully elucidated [19]. Strong evidence supports the involvement of (neuro)inflammation in the pathophysiology of certain headache types, including MiG [20,21,22,23], while the role of inflammatory mediators in TTH is inconclusive [23]. The exact pathogenesis of MiG remains unclear [24], but limited evidence suggests a possible protective role of MiG in relation to BC. Some studies have even suggested a potential association between a migraine and a favorable prognosis in diagnosed BC patients [25,26,27,28,29,30]. Female reproductive hormones might partially explain the complex mechanism linking MiG to BC [31], even if the hypothesis of an interplay between headache and a BC prognosis remains controversial [32,33].

The aim of this study is to explore the brain metabolic patterns in BC patients undergoing [^18^F]FDG PET imaging as part of the staging procedures during neoadjuvant chemotherapy (NAC). To this scope, [^18^F]FDG brain metabolism was compared among BC patients with TTH, MiG, and those without headache. Secondarily, we evaluated whether the diagnosis of primary headache forms, and the corresponding [^18^F]FDG PET brain metabolic pattern, was associated with the BC response to NAC.

## 2. Materials and Methods

### 2.1. Patient Population

Patients diagnosed with BC referring to the Breast Unit of the IRCCS Humanitas Research Hospital were screened and invited to participate in this observational prospective proof-of-principle study. Inclusion criteria were female gender, age ≥18 years, histologically confirmed diagnosis of BC, I to III clinical stage, and candidates for neoadjuvant chemotherapy (NAC) as per the standard of care. NAC consisted of a 3-month anthracycline-based chemotherapy followed by an additional 3-month taxane-based chemotherapy. Specifically, patients with triple-negative breast cancer received weekly carboplatin plus paclitaxel, those with human epidermal growth factor receptor 2 (HER2)-positive breast cancer received docetaxel plus trastuzumab, and patients with luminal-like disease received docetaxel alone. All patients signed informed consent before entering the study. We included in the present analyses all patients prospectively recruited from July 2019 to July 2022 who performed a staging and/or restaging total-body [^18^F]FDG PET/CT with a dedicated brain scan. Patients with incomplete data of either BC or headache, and patients with metastatic BC, were excluded from the cohort. Demographic data, menopausal status, and histopathological tumor characteristics were recorded for each patient. BC molecular subtypes were defined based on immunohistochemistry biomarkers, including estrogen receptor (ER), progesterone receptor (PgR), and human epidermal growth factor receptor 2 (HER2). Patients were categorized into two main groups: HER2-negative hormone receptors (HR)-negative disease (namely triple-negative BC, or TNBC) and HER2-positive BC. The HER2-positive group encompassed the “HER2” subtype (HER2+ and HR−) and the “LUMHER” subtype (HER2+ and HR+). An ICHD-3 criteria-based questionnaire investigating the type, site, duration, and intensity of the pain and a list of accompanying symptoms was filled to diagnose the headache type and collect information about episodes (see Table S1 in Supplementary Material for detailed criteria). The age of headache onset, number of monthly headache days, episodic or preventive treatments for headache, as well as status of activity of the headache were recorded. A headache-experienced neurologist assessed the reliability of responses to the questionnaires. Accordingly, patients were classified into three groups: TTH patients, MiG headache patients, and individuals without headache. None of the patients reported symptoms compatible with trigeminal autonomic cephalalgia. For all patients, data about pathological treatment response assessed in the breast surgical specimen removed after neoadjuvant therapy were collected, and this information was used to accordingly group the patients into two categories: pathological complete response (pCR) and non-complete pathological response (non-pCR). pCR was defined as the disappearance of invasive cancer both in the breast and in the axilla. All procedures were conducted in accordance with the Declaration of Helsinki and were approved by the IRCCS Humanitas Research Hospital Ethics Committee (protocol identifying number: ONC/OSS-02/2019).

### 2.2. PET Acquisition and Image Analyses

Glucose levels were checked in fasting patients (at least 6 h) and an intravenous cannula was positioned prior to [^18^F]FDG administration. Patients were positioned lying in a quiet dark room with their eyes closed; afterwards, the intravenous injection of [^18^F]-FDG (~6 MBq/kg) was performed. PET/CT images were acquired approximately 60 min after radiopharmaceutical administration following the EANM guidelines [34,35] with an integrated GE Discovery PET/CT 690 equipped with LYSO crystals and a 64-slice CT scanner (General Electric Healthcare, Waukesha, WI, USA). Images were acquired and reconstructed for all patients using the same protocol. Details about image acquisition and reconstruction parameters are provided in Table S2 of Supplementary Material.

DICOM files were exported and converted to the Analyze format with Mango software (Research Imaging Institute, UTHSCSA). A visual quality control of the brain PET images was performed for excluding subjects with imaging artefacts in the brain (e.g., acquisition or reconstruction issues and excessive patient motion with respect to the CT) from the statistical analysis.

Prior to any statistical analysis, PET scans were spatially preprocessed with Statistical Parametric Mapping (software version 12—SPM12) [36] software, following a spatial normalization and smoothing procedure. The [^18^F]FDG PET images of the subjects were normalized with the template developed by Della Rosa and co-authors [37], and are available to download in the “Templates” section (on the SPM official website, https://www.fil.ion.ucl.ac.uk/spm/ext/#tpl accessed on 1 February 2022). Other estimation options for spatial normalization, including source image smoothing (8), affine regularization (ICBM space template), and nonlinear frequency cut-off (25), nonlinear iterations (16), and nonlinear regularization (1), were kept equal to the default batch variables of SPM12. Spatially normalized images of all subjects were subsequently smoothed with an isotropic 3D Gaussian kernel of 8 mm FWHM before entering the statistical analysis.

### 2.3. Statistical Analysis

Frequency tables and descriptive statistics (percentage, mean, median, range, and standard deviation) were used to summarize patient characteristics. All preprocessing and statistical analysis steps were performed using SPM12 [36] running in Matlab R2021b. Accordingly, a whole brain voxel-wise assessment was carried out both at the single patient and group level. This choice was due to the proof-of-concept nature of the study, thus allowing us to evaluate brain metabolic correlates of headache (and related subtypes) without any anatomical or functional a priori hypothesis.

#### 2.3.1. Population Comparative Analysis

At first, we tested the statistical differences amongst the entire group of BC patients, including brain PET images both before and after chemotherapy. In detail, we applied the two-sample *t*-test implemented in the SPM for comparing two independent groups. The effects of neoadjuvant chemotherapy on breast cancer patients were assessed by comparing their brain images before and after the treatment. To perform the two-sample *t*-test in SPM, we first preprocessed the brain images of BC patients, applying standard normalization and smoothing procedures to ensure accurate spatial alignment and noise reduction. Next, we defined groups for the comparison and performed a voxel-wise application across the whole brain. This process yielded statistical parametric maps that represent areas of the brain where there are significant differences in brain activity or structure between the pre-chemotherapy and post-chemotherapy conditions.

##### Metabolic Correlates in Primary Headache Types

A voxel-wise analysis (Two-sample *t*-test in SPM) was performed to compare brain metabolism among BC patients with TTH, MiG, and without episodes of headache and based on various concomitant clinical variables as detailed in the Supplementary Material. A *p*-value < 0.001, corrected with the family-wise error (FWE) option at the cluster level, was accepted as significant when not otherwise specified.

A further comparison among brain metabolism of TTH, MiG, and no-headache subjects was made by considering treatment response to NAC. Pathology was used as a reference standard and the pCR was the clinical endpoint. The frequencies of pCR and non-pCR were considered depending on the presence of headache and headache subtypes.

##### Tumor Subtype

BC patients were further categorized into two groups based on their tumor subtype and changes in the brain metabolism were tested before and after NAC. Correlations of brain metabolism patterns with different covariates were also assessed using a multiple regression model. The significance of the correlation test was established at a *p*-value < 0.02, corrected with the family-wise error (FWE) option at the cluster level.

#### 2.3.2. Single-Subject Analysis

The evidence of a different behavior between pre- and post-neoadjuvant chemotherapy in the brain metabolism was studied in detail in BC patients who underwent imaging at both baseline and for restaging. In this case, we compared the brain uptake of each patient before and after NAC to determine specific changes in the brain metabolism. Single-subject analyses were performed, comparing each subject with respect to a healthy control (HC) group (AIMN dataset, FDG BRAIN NORMAL DATASET) [38] using the single sample *t*-test in SPM. The statistical significance was established at a *p*-value < 0.02, corrected with the family-wise error (FWE) at the cluster level.

## 3. Results

**Figure 1:**
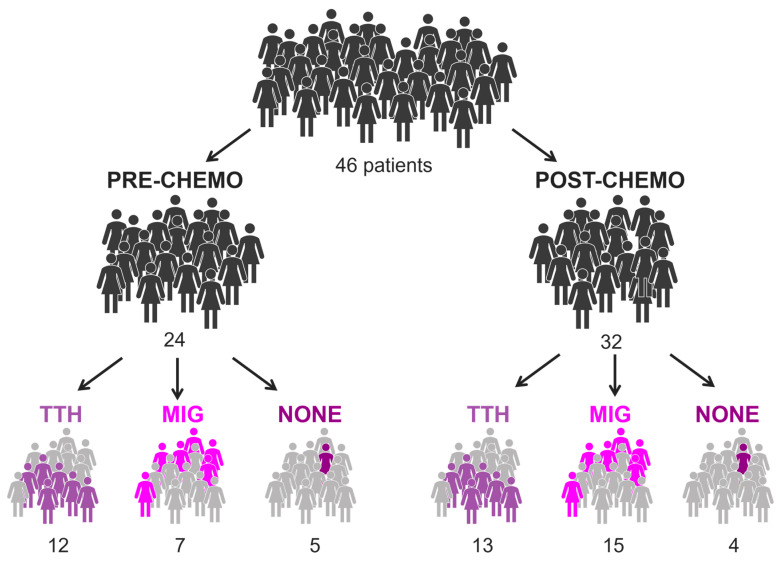
Illustration of BC population, considering treatment and presence of headache. In total, 10 of 46 patients performed both pre- and post-chemo exam.

Figure 1 and Table 1 summarize the main characteristics of patients included in the analysis. Primary headache was diagnosed in 39/46 patients (85%).

All the patients diagnosed with headache experienced headaches before the diagnosis of BC and the initiation of NAC. We did not observe any changes in headache characteristics during the course of NAC treatment.

### 3.1. Population Comparative Analysis

No statistically significant differences were observed among BC patients based on their clinical baseline characteristics. We divided the sample into subgroups based on the headache diagnosis (TTH, MiG, and non-headache) and BC subtype (TNBC, HER2, and LUMHER2) to examine potential disparities in brain metabolism among each subgroup. Additionally, we assessed differences in PET scans before and after NAC to exclude possible chemotherapy-related PET changes.

#### 3.1.1. Metabolic Correlates in Primary Headache Types

**Figure 2:**
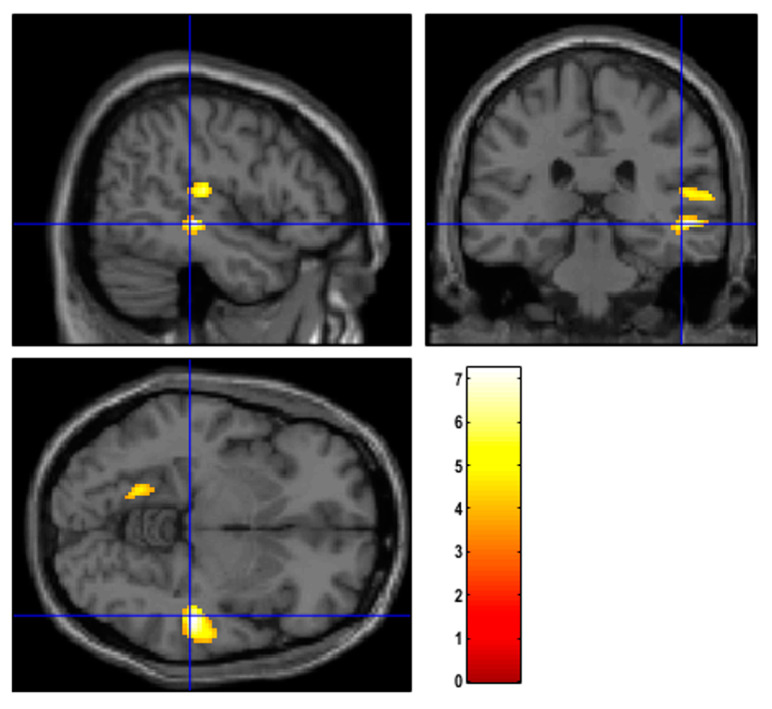
Regions of hypometabolism identified with SPM12 in patients suffering from TTH with respect to MiG before NAC. Examining all of the voxels in the brain and testing each voxel individually at a p-threshold of 0.001, we controlled the cluster-level family-wise error rate.

Patients with TTH exhibited hypometabolism in the right temporal lobe and in the insular region compared to both MiG patients (Figure 2) and those without headache (Figure S1) (*p*-value = 0.001; cluster-level family-wise error rate (FWEc) = 279). However, this hypometabolic pattern was observed, specifically, in patients prior to receiving NAC, while it was not evident after NAC. No significant differences in brain metabolism were found between TTH, MiG, or subjects without headache in the restaging scan.

Differences were observed in BC patients with TTH in terms of ER and PgR positivity and menopausal status (see Figure S2 of Supplementary Material for details). No statistically significant correlations were found between MiG and clinical variables.

**Figure 3:**
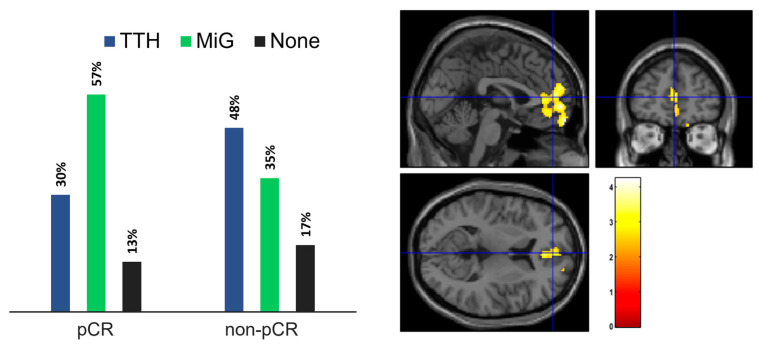
(**left**) Headache presence in patient subgroups according to response to NAC (**right**). Statistical map of the two-sample *t*-test performed between TTH and MiG subjects before chemotherapy treatment. Examining all of the voxels in the brain and testing each voxel individually at a p-threshold of 0.02, we controlled the cluster-level family-wise error rate.

Regarding pathology, 23 subjects achieved a pCR to NAC, while 22 did not (non-pCR). One patient discontinued neoadjuvant treatment and thus was excluded from this specific analysis. As illustrated in Figure 3 (left panel), amongst the pCR group, 7 subjects had TTH (30%), 13 had MIG (57%), and 3 did not experience any form of headache (13%). The non-pCR group included 11 patients with TTH (48%), 8 with MiG (35%), and 4 without headache (17%).

To investigate the anomalies in brain metabolism in the restaging PET, we compared patients based on NAC response and headache type. Among TTH patients, those with a pCR showed hypermetabolic brain regions in the bilateral anterior medial frontal cortex (*p*-value = 0.02; FWEc = 1299), as illustrated in Figure 3 (right panel). No significant differences were found in MiG patients. Due to the limited number of subjects, a similar comparison was not conducted for individuals without headache.

#### 3.1.2. Tumor Subtype

One group consisted of TNBC subjects, while the second group contained the HER2 and LUMHER2 subjects. The two-sample *t*-test model showed no statistical differences in both groups when comparing brain metabolism before and after the treatment. When baseline and restaging PET images were compared between the two groups, differences were obtained from the two-sample *t*-test model. Before NAC, TNBC patients presented hypometabolic brain regions located in the medial parietal cortex (precuneal region) (*p*-value = 0.001; FWEc = 295) with respect to the HER2/LUMHER2 group (Figure S3, left). After neoadjuvant therapy, the behavior was inverted, and the TNBC involved hypermetabolic brain regions in the left cerebellar cortex (*p*-value = 0.002; FWEc = 389) with respect to the other group (Figure S3, right). 

### 3.2. Single-Subject Analysis

**Figure 4:**
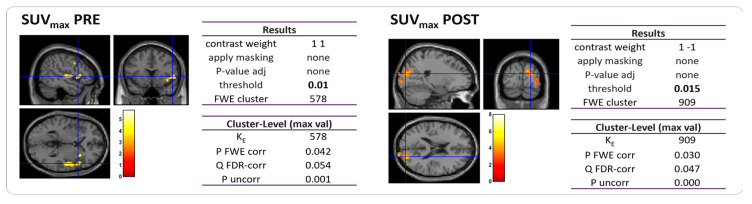
Statistical map of the two-sample *t*-test performed between tumor SUV_max_ and brain metabolism prior to (**left**) and post (**right**) NAC. Examining all of the voxels in the brain and testing each voxel individually at a p-threshold of 0.01, we controlled the cluster-level family-wise error rate.

Within this group, 7 out of 10 patients were diagnosed with TTH, one suffered from MiG, and two had no headache. For these 10 patients, we examined the correlation between brain metabolism and their primary tumor uptake, quantified as the maximum standardized uptake value (SUV_max_) in PET scans, as well as their response to chemotherapy. We found a direct correlation between tumor SUV_max_ and brain metabolism in [^18^F]FGD PET images before chemotherapy, while an inverse correlation was found between these variables after NAC (Figure 4). In other words, patients with an increased brain metabolism before chemotherapy showed high SUV_max_, particularly in the right insular cortex (*p*-value = 0.01; FWEc = 578), while on the other hand, a high brain uptake was associated with low SUV_max_ after chemotherapy. Similar findings were observed when assessing the correlation between the delta-maximum standardized uptake value (ΔSUV_max)_ and brain metabolism, indicating that patients with higher hypermetabolism had a greater variation in the tumor uptake of the [^18^F]FDG (*p*-value = 0.015; FWEc = 909).

Within this group of 10 patients, we conducted a detailed analysis of their single brain metabolism before and after NAC, also comparing abnormalities at the cluster level. We found that only five subjects had hypometabolic brain regions before receiving chemotherapy, compared to a control group of healthy patients. On average, the number of voxels showing hypometabolism with respect to the HC group was k_E_ = (4.2 ± 1.9) ‰. These patients had different tumor subtypes, hormone receptor expression, ki-67 levels, and menopausal status. After NAC, all BC patients no longer exhibited hypometabolic brain regions and were statistically similar to the HC group, up to a ***p***-value equal to 0.05.

## 4. Discussion

In our cohort of BC patients, almost all subjects suffered from a primary form of headache. A high incidence of headache episodes in BC patients has been widely reported, including a recent study that examined a large cohort of BC patients [33]. Furthermore, a review by Wolff et al. [39] on adverse events from placebo arms of randomized studies revealed that headache is more common among BC patients compared to other types of cancer. Notably, headache in our cohort of TTH/MiG patients came first relative to the diagnosis of BC and the initiation of NAC, and NAC treatment did not affect it. These findings are consistent with our previous study, where we demonstrated that systemic therapies for breast cancer, including chemotherapy, did not significantly influence headaches when considering the entire duration of the treatment [33]. Despite the inverse correlation between MiG and BC risk reported by some studies [25,26,28,40] and according to others [27,32], we observed a slightly higher prevalence of MiG in our BC patient population compared to TTH and it was even more prevalent with respect to non-headache patients (46% vs. 39% vs. 15%, respectively). Nonetheless, the heterogeneity of our cohort enabled us to further investigate possible interconnection between the BC clinical outcome and headache diagnosis by studying differences in [^18^F]FDG brain uptake amongst different groups.

Firstly, we compared the brain metabolic correlates among different headache types. TTH subjects exhibited hypometabolism in the right temporal lobe and in the right insula at baseline, and these findings were also confirmed in the single-subject analyses. Interestingly, contrary to findings in the literature, the MiG patients of our cohort did not show any hypometabolism with respect to the other groups, either before or after NAC. Recently, Torres-Ferrus et al. [12] performed [^18^F]FDG PET and MRI in episodic and chronic MiG patients, as well as in healthy controls, and found a statistically significant bilateral temporal pole hypometabolism in both chronic and episodic MiG patients compared to controls, with a better definition in chronic forms and an intermediate alteration in episodic ones. The absence of brain abnormalities in our MiG cohort was unexpected. One plausible explanation for this outcome could be the limited number of headache days per month reported by our cohort. Indeed, we found a lower frequency of headaches per month in our cohort (about 2 days for MiG) compared to other studies [12]. According to Torres-Ferrus et al. [12], the higher the number of headache days, the more pronounced the alterations in brain metabolism. Therefore, it is possible that brain metabolism alterations induced with MiG episodes were not impactful enough to be discriminated from those of subjects without headache using [^18^F]FDG PET imaging.

It should be acknowledged that the brain metabolic responses in a migraine are not yet fully elucidated and cannot be entirely supported with the available fluid biomarkers related to brain metabolism. Nonetheless, our results aligned with some experiences reported in the literature. Altered levels of certain metabolites, such as cholesterol, glucose, pyruvate, and specific amino acids (leucine, isoleucine, methionine, valine, proline, and serine), have been found in subjects with a migraine [41]. These amino acid alterations, along with tryptophan and serotonin hypometabolism, have been implicated in determining detectable changes in a migraine brain [42]. These emerging findings appear to be robust, particularly considering the key role of serotonin alterations in migraine pathophysiology, as supported with blood and neurophysiological studies [43,44]. While our study contributes valuable [^18^F]FDG PET data, it is clear that further investigations are required to comprehensively understand the complexities of brain metabolic responses in a migraine. The integration of various biomarkers and metabolic pathways may shed more light on the underlying mechanisms involved in migraine physiopathology.

To the best of our knowledge, this is the first study investigating the metabolic correlates of TTH using [^18^F]FDG PET within a cohort of BC patients. While it can be speculated that hypometabolism in the right insular areas may represent an [^18^F]FDG PET trademark of TTH, other possible pre-existing or concomitant conditions, including BC itself, should be considered [45,46,47,48,49,50,51,52,53]. In the first sense, in our sample of BC patients, TTH subjects were more affected by headache days and exhibited PET activity similar to that observed in MiG patients with a higher headache burden. In the other sense, the right insular activity seems to be heavily influenced by BC in two manners. First, the hypometabolism in the right limbic areas was not observed in subjects who underwent a post-chemotherapy PET analysis, suggesting that systemic chemotherapy can directly or indirectly alter brain metabolism through the interaction with the breast neoplasm. This result is in accordance with recent reports on a large cohort of non-metastatic BC patients after NAC [54]. Schroyen et al. [54] observed both hyper- and hypometabolism in different brain regions depending on the administered regimen of chemotherapy for BC, even in the absence of any overt neurological symptoms, such as cognitive impairments. Second, the baseline PET pattern in the right insular regions appeared to be correlated to BC activity. The level of breast hypermetabolism was also associated with hypermetabolism in the right insula, and the latter was related to a larger variation in the BC pathological response after NAC.

Notably, our results suggest an association between primary headache and treatment response to NAC. Specifically, in the MiG group of patients, we observed a higher rate of pCR compared to non-pCR (57% vs. 36%), while in the TTH group, we observed an inverse trend with a higher prevalence of non-pCR compared to pCR (50% vs. 30%). Interestingly, despite the small number of patients, we noticed a similar prevalence of pCR and non-pCR in the no-headache group (13% vs. 18%). Collectively, our findings suggest that patients with MiG may have a more favorable prognosis than TTH and no-headache patients since a pCR to NAC has been reported as a surrogate endpoint for long-term event-free and overall survival of BC [55,56].

Due to the cross-sectional nature of the study, we cannot exclude that this TTH could not be a pure primary headache and instead represent a TTH-like headache developed in BC patients and that it could also be influenced to some extent by BC itself, possibly though an enhanced pro-inflammatory susceptibility involving alterations in the right insular brain regions. To support this last hypothesis, we also found that BC appears to selectively influence brain [^18^F]FDG metabolic activity in TTH patients. Specifically, we observed a direct correlation between brain metabolism and a high level of HR (Supplementary Material). High levels of ER and PgR in BC patients before NAC were found to correlate with the hypermetabolic uptake of the right and left parietal lobe, respectively. The association between headache and female hormonal levels has been reported in both MiG and TTH populations [22,57] and has its rationale in the diffuse distribution of ER in the cortex [58].

Another relevant finding related to female hormone levels was the menopausal status of the patients. Post-menopausal TTH patients exhibited hypometabolic regions in the left cerebellar cortex compared to pre-menopausal individuals. It is worth noting that we did not observe any difference between pre- and post-menopausal woman in the MiG group. Nevertheless, the limited number of post-menopausal patients with MiG in our cohort (6 against 11 post-menopausal TTH patients) may have limited the statistical significance of this test. As an independent explanation for this result, it should be mentioned that an inverse relationship between brain glucose metabolism and dysmetabolic states (e.g., obesity and type 2 diabetes), which occur more frequently in post-menopausal women, has been described [51,52,53]. However, none of our patients had type 2 diabetes, but we observed a slightly higher number of overweight patients in the TTH group compared to the MiG group, where the majority of patients had a healthy weight range.

Some limitations of the present study are worth mentioning. Due to its exploratory nature, this study did not have an a priori sample size calculation, so this could have hampered the chances of obtaining significant results, such as those in the MiG group. However, our study has a significant implication for future research that should delve into the intricate pathophysiological interactions between breast cancer and the nervous system. Another possible bias could have been derived with the use of a questionnaire instead of a formal amnestic interview and with the cross-sectional design to correlate results with possible changes in the headache frequency. However, we observed that sudden changes in headache frequency are not typical in BC patients at the diagnosis or during chemotherapy. Moreover, the use of [^18^F]FDG PET/CT performed during BC staging/restaging offers the opportunity to objectively investigate brain metabolism and the interplay between headache and BC as part of routine examinations without incurring additional costs.

## 5. Conclusions

This study was performed on a small yet homogenously selected group of BC patients—candidates to preoperative chemotherapy. By means of [^18^F]FDG PET/CT, which is routinely performed in this oncological setting, we extensively explored interactions between BC, headache, and NAC. Although we employed a self-administered headache questionnaire that may introduce some bias, the use of functional brain PET provided an ‘objective quantifier’ that partially mitigated this limitation. Our preliminary findings demonstrate alterations in brain metabolism, particularly in the TTH patient group. Furthermore, they reveal interactions between the primary tumor, patients’ characteristics, and TTH, confirming the complexity of the underlying physiopathological mechanisms, hypothetically related to both the tumor environment and the hormonal status of the patients. This raises the hypothesis of a BC-related TTH-like headache. It is crucial to confirm our results in a large prospective cohort of patients. Overall, our results contribute to understanding the interactions between breast cancer and the nervous system, providing some crucial insights on how this interplay may impact on pathological response to NAC. By gaining further insights into these complexities, we can potentially optimize the effectiveness of preoperative chemotherapy treatment, leading to improved patient prognosis.

## Figures and Tables

**Table 1:** Demographic and clinical baseline characteristics of patients included in the cohort.

	Population	TTH	MIG	No Headache
Population (n (%))	46	18 (39%)	21 (46%)	7 (15%)
Age (mean)	50	52	48	53
stdv	11	9	12	16
median	52	52	47	55
range	27–77	27–65	30–72	33–77
Hormone Receptor				
Estrogen (n and mean ± stdv)				
ER < 20%	28 1.4 ± 3.6	11 2.1 ± 3.3	14 1.1 ± 4.0	3 2.5 ± 4.3
ER > 20%	18 76.4 ± 19.4	7 80.0 ± 11.2	7 68.6 ± 21.1	4 83.8 ± 12.5
Progesteron (n and mean ± stdv)				
PgR < 20%	33 0.4 ± 1.1	14 0.5 ± 1.5	14 0.1 ± 0.3	5 0.8 ± 1.8
PgR > 20%	13 (28%) 61.5 ± 26.3	4 46.3 ± 27.2	7 65.7 ± 27.5	2 77.5 ± 3.5
Ki-67 (n (%) and mean ± stdv)				
Ki-67 < 20%	11 16.7 ± 3.1	6 15.9 ± 3.5	5 17.6 ± 2.5	0 -
Ki-67 > 20%	35 49.7 ± 20.2	12 47.6 ± 21	16 52.2 ± 18.3	7 47.5 ± 25.2
Tumor Subtype (n (%))				
TNBC	18 (39%)	7 (39%)	8 (44%)	3 (17%)
HER2+	8 (17%)	4 (50%)	4 (50%)	0 (0%)
LUMHER2	20 (43%)	7 (35%)	9 (45%)	4 (20%)
Menopause (n (%))				
Premenopausal	21 (43%)	5 (24%)	13 (62%)	3 (14%)
Perimenopausal	4 (11%)	2 (40%)	2 (40%)	0 (0%)
Postmenopausal	21 (46%)	11 (52%)	6 (29%)	4 (19%)
BMI (n (%))				
<18.5	6 (13%)	2	2	2
18.5–23.8	20 (44%)	6	11	3
23.9–28.6	15 (33%)	8	6	1
28.7–34.9	3 (6%)	1	1	1
35–39.9	1 (2%)	0	0	1
>40	1 (2%)	1	0	0
Outcome (n (%))				
pCR	23 (50%)	7 (30%)	13 (57%)	3 (13%)
non-pCR	22 (48%)	10 (45%)	8 (36%)	4 (18%)
Not available	1 (2%)	1 (100%)	0 (0%)	0 (0%)
Headache (mean ± std)				
Frequency *	1.7 ± 2.1	0.8 ± 0.7	2.0 ± 2.7	0.0 ± 0.4
[^18^F]FDG PET/CT (n (%))				
Pre-chemotherapy	14 (30%)	5	6	3
Post-chemotherapy	22 (30%)	6	14	2
Both	10 (22%)	7	1	2

* Number of days per month of headache episode. [^18^F]FDG: 2-deoxy-2-[^18^F]fluoroglucose; PET: positron emission tomography; TNBC: triple-negative breast cancer; HER2+: human epidermal growth factor receptor 2-positive; LUMHER2: luminal HER2+ human epidermal growth factor receptor 2-positive; TTH: tension-type headache; MIG: migraine; BMI: Body Mass Index; CR: complete response.

## Data Availability

The manuscript represents valid work, and neither this manuscript nor one with substantially similar content under the same authorship has been published or is being considered for publication elsewhere. Raw data are available on specific request to the corresponding author.

## Supplementary Materials

The following supporting information can be downloaded at: https://www.mdpi.com/article/10.3390/cancers15164147/s1, Table S1: Diagnostic ICHD-3 criteria for tension-type headache (TTH) and migraine (with and without aura); Table S2: Image acquisition and reconstruction parameters; Figure S1: Regions of hypometabolism in patients suffering from TTH with respect to patients without headache before NAC; Figure S2: Statistical map of the two-sample *t*-test performed for TTH patient group considering ER (left) and PgR (right) receptor status. Figure S3: Statistical map of the two-sample *t*-test performed between TNBC and HER2 patient groups prior to (left) and after (right) NAC.
