# Supplementary material for: Imaging Correlates between Headache and Breast Cancer: An [18F]FDG PET Study
Source: Cancers (Basel). 2023 Aug 17;15(16):4147. doi: 10.3390/cancers15164147 (PMC10453040; doi:10.3390/cancers15164147)
Supplement: Supplementary file 1 [file cancers-15-04147-s001.zip › cancers-2481365-SI.pdf]

Supplementary material

Imaging Correlates between Headache and Breast Cancer: An [^{18}F]FDG PET Study

Lidija Antunovic [1,†,‡], Alessia Artesani [1,2,†], Alessandro Viganò [3], Arturo Chiti [1,2,‡,§], Armando Santoro [1,2], Martina Sollini [1,2,*], Silvia D. Morbelli [4,5] and Rita De Sanctis [1,2]

1 IRCCS Humanitas Research Hospital, Via Manzoni 56, 20089 Rozzano, Italy; antunovic.lidija@hsr.it (L.A.); alessia.artesani@hunimed.eu (A.A.); chiti.arturo@hsr.it (A.C.); armando.santoro@cancercenter.humanitas.it (A.S.); rita.de_sanctis@hunimed.eu (R.D.S.)
2 Department of Biomedical Sciences, Humanitas University, Via Rita Levi Montalcini 4, 20072 Pieve Emanuele, Italy
3 Neurology, Fondazione Don Gnocchi, 20162 Milan, Italy; avigano@dongnocchi.it
4 Nuclear Medicine Unit, IRCCS Ospedale Policlinico San Martino, 16132 Genoa, Italy; silviadaniela.morbelli@hsanmartino.it
5 Department of Health Sciences, University of Genoa, 16132 Genoa, Italy
* Correspondence: martina.sollini@hunimed.eu; Tel.: +39-028-224-5614
† These authors contributed equally to this work.
‡ Currently address: Department of Nuclear Medicine, IRCCS Ospedale San Raffaele, 20132 Milan, Italy.
§ Currently address: Faculty of Medicine and Surgery, Vita-Salute San Raffaele University, 20132 Milan, Italy.

Table S1. Diagnostic ICHD-3 criteria for tension-type headache (TTH), and migraine (with and without aura) [1].

Tension-type headache	A. At least ten episodes of headache occurring on 1–14 days/month on average for > 3 months (≥ 12 and < 180 days/year) and fulfilling criteria B-D<br>B. Lasting from 30 min to 7 days<br>C. At least two of the following four characteristics:<br>1. Bilateral location<br>2. Pressing or tightening (non-pulsating) quality<br>3. Mild or moderate intensity<br>4. Not aggravated by routine physical activity such as walking or climbing stairs<br>D. Both of the following:<br>1. No nausea or vomiting<br>2. No more than one of photophobia or phonophobia<br>E. Not better accounted for by another ICHD-3 diagnosis
Migraine without aura	A. At least five attacks1 fulfilling criteria B-D<br>B. Headache attacks lasting 4–72 h (untreated or unsuccessfully treated)<br>C. Headache has at least two of the following four characteristics:<br>1. Unilateral location<br>2. Pulsating quality<br>3. Moderate or severe pain intensity<br>4. Aggravation by or causing avoidance of routine physical activity (e.g., walking or climbing stairs)<br>D. During headache, at least one of the following:<br>1. Nausea and/or vomiting<br>2. Photophobia and phonophobia<br>E. Not better accounted for by another ICHD-3 diagnosis
Migraine with aura	A. At least two attacks fulfilling criteria B and C<br>B. One or more of the following fully reversible aura symptoms:<br>1. Visual<br>2. Sensory<br>3. Speech and/or language<br>4. Motor

	5. Brainstem<br>6. Retinal<br>C. At least three of the following six characteristics:<br>1. At least one aura symptom spreads gradually over ≥ 5 min<br>2. Two or more aura symptoms occur in succession<br>3. each individual aura symptom lasts 5–60 min<br>4. At least one aura symptom is unilateral<br>5. At least one aura symptom is positive<br>6. The aura is accompanied, or followed within 60 min, by headache<br>D. Not better accounted for by another ICHD-3 diagnosis

Table S2: Image acquisition and processing parameters			
Imaging		3D PET, CT	
Field of view – region of interest		Brain	
Patient preparation		Fasting for at least 6 hours before injection; Blood glucose levels below 200 mg/dl were requested.	
Radioactive tracer		2-Deoxy-2-[^{18}F]fluoroglucose ([^{18}F]FDG); Intravenous administration<br>Injected activity range (350-550 MBq)<br>Uptake time range 55-65 minutes with patient lying in a quiet dark room with eyes closed	
Contrast agent		None	
Acquisition and reconstruction		Discovery 690 – GE	
		PET	CT
	Acquisition mode, time	List mode, 10 minutes	-
	Crystal	LYSO	-
	Reconstruction	Iterative (OSEM), time of light (TOF) with VUE point FX (3.0 mm), FOV of reconstruction 30	-
	Attenuation correction	CT	-
	Matrix (pixels)	256x256	512×512
	Resolution (mm)	2.73×2.73	1.37×1.37
	Slice thickness (mm)	3.27	3.27

	Slices	-	64
	Voltage (kV)	–	140
	Tube current (mA)	-	130

S.1 Results

To explore the meaning of metabolic pattern in BC patients with different primary headache types, we correlated brain metabolism with relevant clinical variables, such as frequency of headache, BC molecular subtype, marker of proliferation (ki-67), ER and PgR expression, and menopausal status.

S.1.1 Metabolic correlates in primary headache types

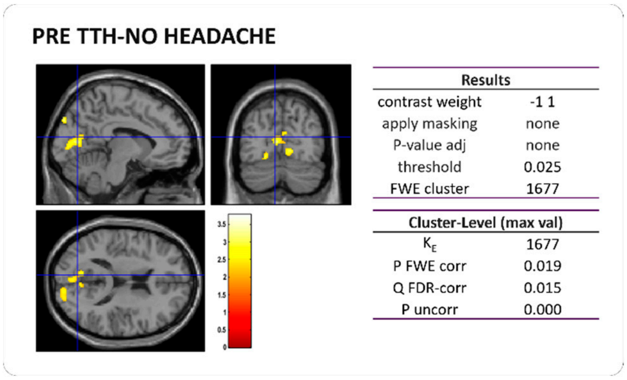

Figure S1 - Regions of hypometabolism in patients suffering from TTH with respect to patients without headache before NAC.

We found a direct correlation between positivity to HR, ER and PgR, and the brain hypermetabolism in patients suffering from TTH (Fig S2). Brain abnormalities located in right superior posterior parietal cortex directly correlates with high level of ER (p-value = 0.015, FWEc = 999), whereas hypermetabolism in the left posterior parietal cortex directly correlated with high level of PgR (p-value = 0.001, FWEc = 231). Moreover, TTH subjects differentiated depending on their menopause status. In fact, we found that post-menopause women suffering of TTH show hypometabolic brain regions located in the left cerebellar cortex with respect to the pre-menopause women with the same headache diagnosis (p-value = 0.001, FWEc = 319). No other direct or indirect correlation was found between brain metabolism and other clinical variables, such as headache frequency and ki-67 value, in TTH subjects. Similar analysis was conducted on MIG subjects, but no statistically significant correlations could be detected in any of the tests.

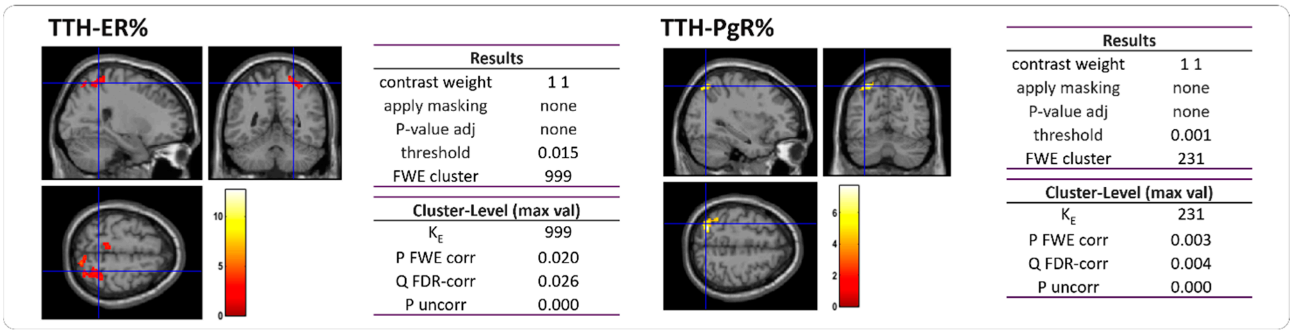

Figure S2 - Statistical map of the two-sample t test performed in TTH patients group considering ER (left) and PgR (right) receptor status.

S.1.2 Tumour subtype

One group consisted of TNBC subjects, while the second group gathered the HER2 and LUMHER2 subjects. The two-sample t-test model showed no statistical differences in both groups when comparing brain metabolism before and after the treatment. When baseline and restaging PET images were compared between the two groups, differences were obtained from the two-sample t-test model. Before NAC, TNBC patients

presented hypometabolic brain regions located in medial parietal cortex (precuneal region) (p-value = 0.001, FWEc = 295) with respect to the HER2/LUMHER2 group (Fig. S3, left). After neoadjuvant therapy, the behaviour was inverted, and the TNBC had hypermetabolic brain regions in the left cerebellar cortex (p-value = 0.002, FWEc = 389) with respect to the other group (Fig. S3, right).

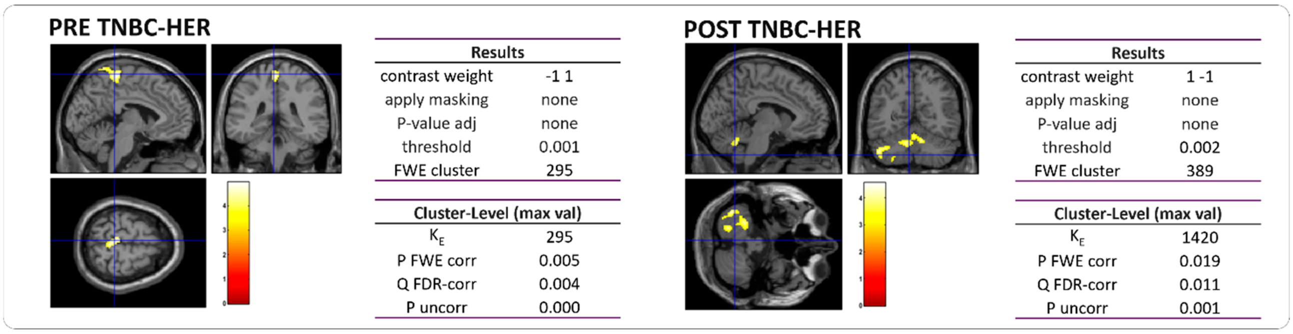

Figure S3 - Statistical map of the two-sample t test performed between TNBC and HER2 patient groups prior (left) and after (right) NAC.

1. Olesen J. Headache Classification Committee of the International Headache Society (IHS) The International Classification of Headache Disorders, 3rd edition. Cephalalgia. 2018;38:1–211.
